# Hydrodynamics of the VanA-type VanS histidine kinase: an extended solution conformation and first evidence for interactions with vancomycin

**DOI:** 10.1038/srep46180

**Published:** 2017-04-11

**Authors:** Mary K. Phillips-Jones, Guy Channell, Claire J. Kelsall, Charlotte S. Hughes, Alison E. Ashcroft, Simon G. Patching, Vlad Dinu, Richard B. Gillis, Gary G. Adams, Stephen E. Harding

**Affiliations:** 1 School of Pharmacy & Biomedical Sciences, Membranes, Membrane Proteins & Peptides Research Group, University of Central Lancashire, Preston, PR1 2HE, United Kingdom; 2 National Centre for Macromolecular Hydrodynamics, School of Biosciences, University of Nottingham, Sutton Bonington, LE12 5RD United Kingdom; 3 Diamond Light Source Ltd., Diamond House, Harwell Science and Innovation Campus, Didcot, Oxfordshire, OX11 0FA, United Kingdom; 4 Astbury Centre for Structural Molecular Biology, University of Leeds, Leeds, LS2 9JT, United Kingdom; 5 School of Health Sciences, University of Nottingham, Nottingham, NG7 2HA United Kingdom

## Abstract

VanA-type resistance to glycopeptide antibiotics in clinical enterococci is regulated by the VanS_A_R_A_ two-component signal transduction system. The nature of the molecular ligand that is recognised by the VanS_A_ sensory component has not hitherto been identified. Here we employ purified, intact and active VanS_A_ membrane protein (henceforth referred to as VanS) in analytical ultracentrifugation experiments to study VanS oligomeric state and conformation in the absence and presence of vancomycin. A combination of sedimentation velocity and sedimentation equilibrium in the analytical ultracentrifuge (SEDFIT, SEDFIT-MSTAR and MULTISIG analysis) showed that VanS in the absence of the ligand is almost entirely monomeric (molar mass *M* = 45.7 kDa) in dilute aqueous solution with a trace amount of high molar mass material (*M* ~ 200 kDa). The sedimentation coefficient *s* suggests the monomer adopts an extended conformation in aqueous solution with an equivalent aspect ratio of ~(12 ± 2). In the presence of vancomycin over a 33% increase in the sedimentation coefficient is observed with the appearance of additional higher *s* components, demonstrating an interaction, an observation consistent with our circular dichroism measurements. The two possible causes of this increase in *s* – either a ligand induced dimerization and/or compaction of the monomer are considered.

Vancomycin is a tricyclic glycopeptide used to treat penicillin-resistant infections caused by Gram-positive bacteria including bloodstream infections and infective endocarditis of enterococcal origin and meningitis caused by methicillin-resistant *Staphylococcus aureus*^1^,^2^. Vancomycin and related glycopeptide antibiotics inhibit the final steps of bacterial cell wall biosynthesis by binding to the C-terminal D-Ala–D-Ala peptide of the muramyl pentapeptide of peptidoglycan precursor Lipid II^3^,^4^,^5^,^6^,^7^,^8^,^9^,^10^. Surface plasmon resonance and other studies revealed values for the equilibrium dissociation constants (K_*d*_) for vancomycin binding of 1.32 μM^11^, 2.7 μM^12^ and 3.39 μM^13^, indicative of moderate binding. Glycopeptide binding results in inhibition of transpeptidase and transglycosylase activities, affecting the crosslinking process in growing peptidoglycan, formation of glycan chains and incorporation of peptidoglycan precursors leading to osmotic shock and cell lysis^14^,^15^,^16^.

Resistance to glycopeptide antibiotics amongst pathogenic bacteria has emerged and spread across the world at a rapid rate^17^,^18^. Of the six types of acquired resistance found in Gram-positive bacteria, the VanA type is the most common amongst clinical enterococci and the first to have spread to staphylococci^19^. Inducible resistance to high levels of vancomycin and teicoplanin (the VanA phenotype) in enterococci occurs as a result of acquisition of genes that encode enzymes that (1) synthesise an alternative target peptidoglycan precursor D-Ala-D-Lac which lacks one of the hydrogen bonding sites involved in glycopeptide binding, thereby constituting a low-affinity precursor that exhibits an approximately 1000-fold reduced affinity for the antibiotic; and (2) eliminate pre-existing high-affinity D-Ala-D-Ala precursors thereby removing the glycopeptide-binding target^20^,^21^. The genes encoding these enzymes are regulated by the VanSR two-component signal transduction system^22^,^23^. VanS is the membrane-bound sensor kinase component involved in inducer sensing and VanR is the partner response regulator component responsible for activating resistance gene expression. *In vitro* experiments with cytosolic VanS of A-type resistance demonstrated both autophosphorylation activity and phosphotransfer activity to VanR^24^. VanR~P increases transcription of the P_R_ promoter involved in VanS/VanR expression thereby increasing its own synthesis, and the P_II_ promoter for the *vanHAX* resistance operon expression^25^. VanR~P is still generated in the absence of VanS due to the activities of acetyl phosphate and/or cross-talking histidine kinase phosphodonors resulting in constitutive activation of the P_R_ and P_II_ promoters^25^. It was suggested therefore that in the absence of glycopeptides, VanS acts as a negative regulator of VanR, removing phosphate from VanR through its phosphatase activity and thus preventing any significant resistance gene expression in the absence of inducer. In the presence of inducer, VanS switches from phosphatase to kinase mode, resulting in higher levels of VanR~P and induction of the *vanHAX* resistance genes. The nature of the activating inducer in VanA-type resistance remains unknown. Studies of distantly-related VanS sensors in streptomycetes and enterococci (VanB-type) have provided evidence that the antibiotic itself or the antibiotic bound to the D-Ala-D-Ala substrate serve as the inducing effectors whilst in VanA-type resistances it has been speculated that the inducer might be a peptidoglycan precursor^26^,^27^,^28^. Here we provide direct *in vitro* evidence that vancomycin binds to the purified A-type VanS and causes changes to the state of the sensor. The study employs an *in vitro* approach developed previously in our laboratory for the routine production of purified intact and active membrane sensor kinases of bacteria including the enterococci^29^,^30^. Heterologously-expressed, His_6_-tagged VanS was purified as an intact membrane protein and verified as intact by mass spectrometry, N-terminal sequencing and Western blotting. In common with most purified intact sensor kinases, it was active in the absence of ligand as revealed using *in vitro* autophosphorylation activity assays^30^. We consider the oligomeric state in aqueous solution (supplemented with 20% glycerol to ensure the protein remains stable and active) using sedimentation velocity (SEDFIT analysis, ref. 31) in the analytical ultracentrifuge supplemented by sedimentation equilibrium measurements, and the routines SEDFIT-MSTAR^32^ and MULTISIG^33^. The solution conformation of the VanS was then analysed using the ELLIPS suite of conformation algorithms^34^ and the effect of the addition of vancomycin was explored.

## Results

### Expression, verification and characterisation of purified active intact VanS

**Figure 1:**
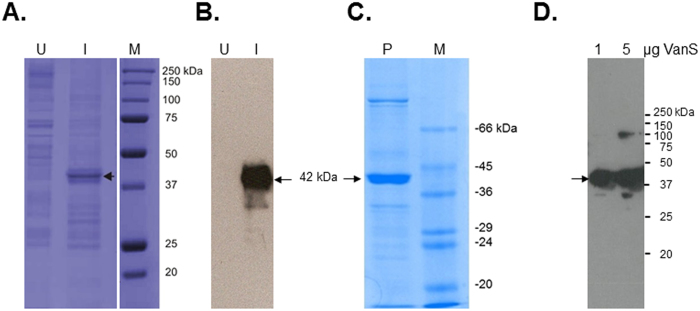
Purification and verification of His_6_-tagged intact VanS. (**A**) SDS-PAGE and (**B**) Western blotting of mixed membrane samples of *E. coli* host cells either uninduced (U) or induced (I) with 1 mM IPTG. (**C**) and (**D**) SDS-PAGE and Western blotting (respectively) of purified intact VanS protein ((**C**) 10 μg in SDS-PAGE; (**D**) 1 and 5 μg in Western blotting). SDS-PAGE employed 4% stacking and 12% resolving gels and proteins were visualised using Coomassie blue staining. Western blotting detected the presence of the C-terminal hexa-histidine tag. Arrows denote the positions of monomeric VanS. (**C**) and (**D**) indicate the presence of multimeric VanS, even under denaturing conditions, as commonly observed during preparation of membrane proteins for SDS-PAGE (see Methods). M, molar mass markers.

**Figure 2:**
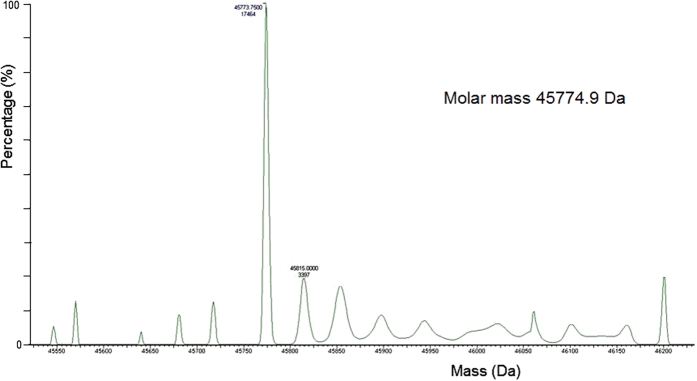
Mass spectrometry of purified VanS. Analysis was carried out using ESI-MS. The m/z spectrum has been converted to a molecular mass profile using Maximum Entropy processing.

**Figure 3:**
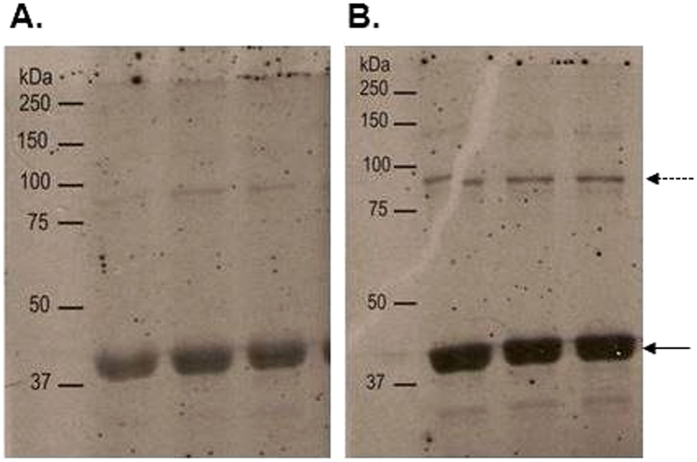
Activity of purified intact VanS. Autophosphorylation assays were performed in buffer with no added detergent (DDM) and in triplicate using 60 pmoles (4 μM) purified VanS incubated at 22.0 °C for 20 min prior to addition of 50 μM ATP (containing 3.75 μCi ɣ-33P ATP) for (**A**) 36 min at 37.0 °C and (**B**) 30 min at 22.0 °C, after which time reactions were stopped by addition of stop buffer as described in Methods. Visualisation of phosphorylated proteins was by separation using SDS-PAGE and autoradiography. Membranes were exposed for (**A**) 8 days and (**B**) 5 days. The solid arrow denotes the position of monomeric VanS; the dashed arrow denotes multimeric VanS.

Expression of the majority of membrane proteins is generally expected to be significantly lower than that of soluble proteins and purification more technically challenging due to the hydrophobicity of membrane proteins. To determine whether VanS was successfully expressed in *E. coli* BL21 [DE3], water lysis preparations of cultures induced or uninduced with 1 mM isopropyl β-D-1-thiogalactopyranoside (IPTG) were prepared as described in Methods. Fig. 1A,B summarises expression in *E. coli* mixed membranes and clearly shows the presence of an additional band corresponding to a molar mass ~42 kDa in induced mixed membranes that is absent in uninduced membranes. Western blotting using a His probe confirmed the 42 kDa protein is His-tagged and absent in uninduced cultures, suggesting that the 42 kDa protein is indeed IPTG-inducible VanS (Fig. 1B). VanS was purified using nickel affinity methods in the presence of *n*-dodecyl β-D-maltoside (DDM) detergent as described previously^35^,^36^. Figure 1C,D shows sodium dodecyl sulphate-polyacrylamide gel electrophoresis (SDS-PAGE) and Western blot analysis of the purified protein which confirms an observed molar mass of approximately 42 kDa for the His-tagged protein relative to the standards, in reasonable agreement with the predicted value taking into account the well-reported challenges of employing this relative technique for integral membrane proteins^35^. To confirm the molecular integrity and identity of purified VanS, electrospray ionisation mass spectrometry (ESI-MS) analysis revealed a mass of (45,775 ± 2) Da which matches closely to the predicted molecular mass for the recombinant intact protein of 45,765 Da (Fig. 2). Furthermore, N-terminal sequencing confirmed the presence of the expected _N_-MNSHM sequence; taken together with the evidence of an intact C-terminus (shown by Western detection of the C-terminal His_6_ tag), it is concluded that VanS is expressed as an intact membrane protein and that the observed lower mass of 42 kDa in SDS-PAGE is attributable to its anomalous migration behaviour by this approximate technique. To confirm that preparations of purified recombinant VanS are in an active conformation, autophosphorylation assays of kinase activity were undertaken as described previously and in Methods^29^,^30^,^36^. Autophosphorylation activity in the presence of ATP was demonstrable, even in the absence of added ligand/signal, as shown to be the case for other active purified intact sensors (Fig. 3)^30^. Therefore, the purified VanS protein used in this study was active, suggesting no evident deleterious effects of the detergent used during the purification on subsequent activities of VanS including binding by exogenously-added ATP. We then explored the oligomeric state in aqueous solution of our preparation of VanS protein using the matrix-free techniques of sedimentation velocity and sedimentation equilibrium in the analytical ultracentrifuge.

### Analytical ultracentrifugation

**Figure 4:**
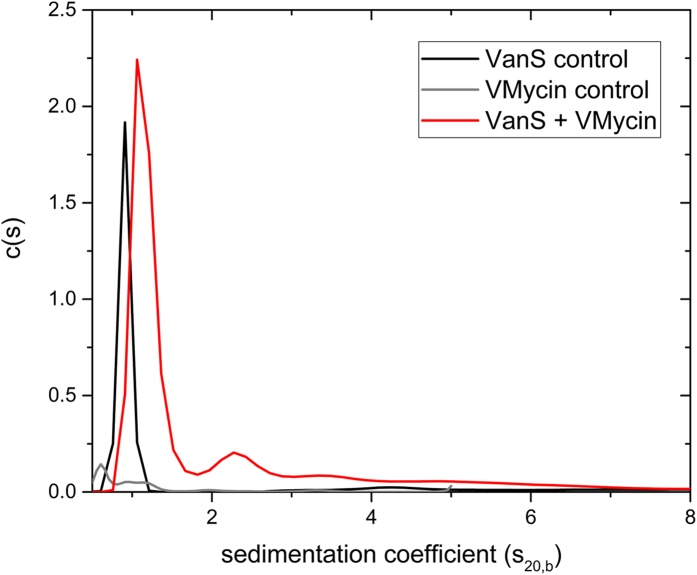
Sedimentation coefficient concentration distribution, *c(s*) vs *s* profile for VanS (Black profile) in HGN buffer pH~7.9, I = 0.1, at 20.0 °C at a loading concentration of 0.25 mg mL^−1^ (5.4 μM) (HGN buffer contains 20% glycerol). Rotor speed = 40000 rpm. Also shown is the profile for vancomycin 0.019 mg mL^−1^ (12.8 μM) (grey profiles) and a mixture of VanS and vancomycin (red profile) under the same conditions. An interaction between the two molecules is clearly evident.

Sedimentation velocity in the analytical ultracentrifuge was performed at 40000 rpm (~120 000 g) at 20.0 °C in HEPES buffer pH~7.9, I = 0.1, lacking detergent (removed by extensive dialysis) and supplemented with 20% glycerol at a low loading concentration of 0.25 mg mL^−1^ to minimize the effects of hydrodynamic non-ideality. SEDFIT c(s) vs s analysis shows the majority of the material is overwhelmingly monomeric in aqueous solution. Figure 4 shows the SEDFIT c(s) vs s distribution (distribution of sedimentation coefficients). The plot shows the solution is highly monodisperse with the main component (96%) of sedimentation coefficient 0.9S and trace amounts (~4%) of a second component at 4.3S. To facilitate comparisons, Table 1 shows also the sedimentation coefficients normalized to standard solvent conditions (namely the density and viscosity of water at 20.0 °C).

**Figure 5:**
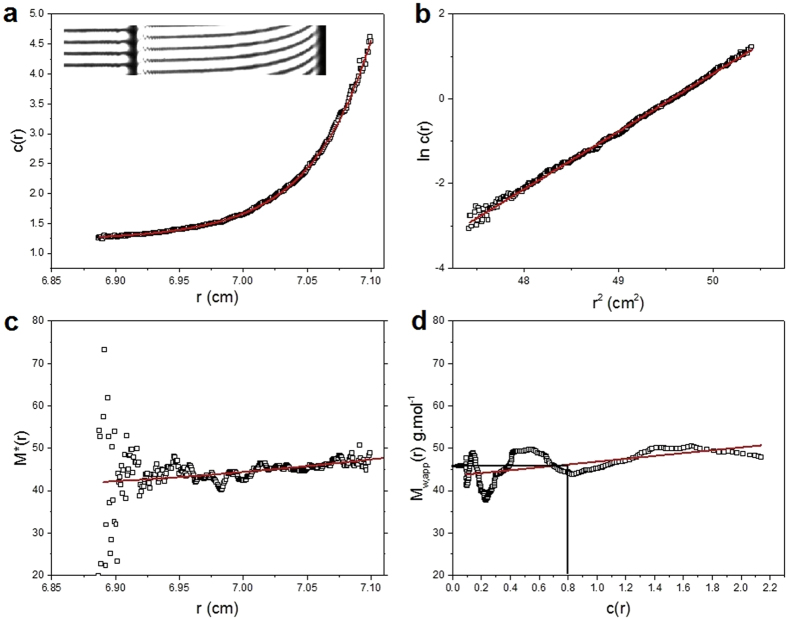
Sedimentation Equilibrium SEDFIT-MSTAR output for analysis of VanS in HGN buffer pH~7.9, I = 0.1, (containing glycerol) at 7.0 °C at a loading concentration of 0.326 mg mL^−1^ in long (20 mm) path length cells. (**a**) concentration (fringe displacement units versus radial displacement from the centre of rotation, r (**b**) log concentration versus the square of the radial displacement (**c**) extrapolation of the M* function to the cell base to yield the “whole distribution” weight average molar mass *M*_*w,*_ = (47.5 ± 1.0) kDa; (**d**) plot of the point average molar mass (local molar mass) – obtained by taking the derivative of the data from plot (b) versus local concentration *c(r*) in the analytical ultracentrifuge cell. The value at the hinge point (where *c(r)* = the cell loading concentration, yields another estimate for the whole distribution *M*_w_ ~ (46.4 ± 1.0) kDa.

**Figure 6:**
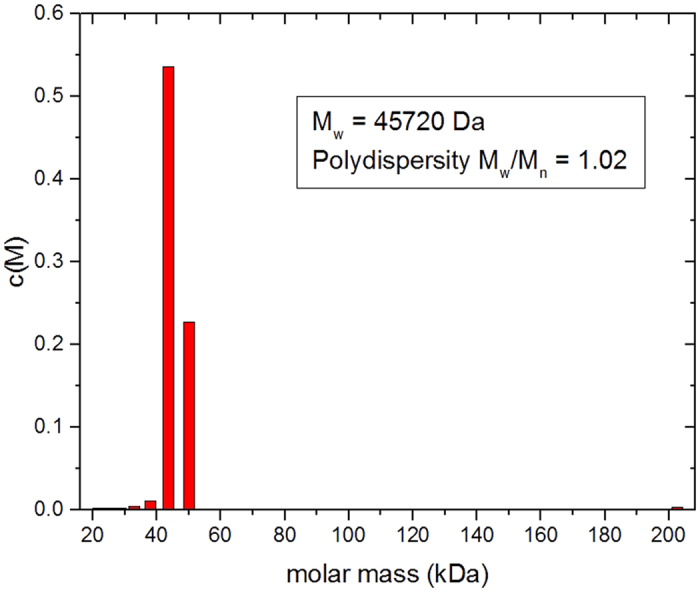
Sedimentation equilibrium Multi-Sig analysis of VanS in HGN buffer pH~7.9, I = 0.1, at 7.0 °C at a loading concentration of 0.3 mg mL^−1^ in long (20 mm) path length cells. The main (monomer) component of *M* = 45.7 kDa is seen (99%) with a trace (1%) amount of a large molecular weight product of *M*~200 kDa, possibly tetramer.

The sedimentation coefficients will not only be influenced by molecular mass but also conformational (frictional) effects. To identify/assign the molecular mass of the species present – and to confirm the main component is the VanS monomer − a further sedimentation equilibrium experiment was performed at a lower rotor speed of 25000 rpm (~48 000 g) and the minimum loading concentration of ~0.3 mg ml^−1^ to minimise the effects of thermodynamic non-ideality. Although not as resolving as sedimentation velocity, sedimentation equilibrium gives a direct measure of molecular weight (molar mass) unaffected by conformation, and for mixtures of more than one component, principally the weight average molar mass. We employ both the M* method of Creeth and Harding^37^ and the ‘Hinge point’ method^32^ implemented in the SEDFIT-MSTAR algorithm of Schuck, Harding and coworkers^32^. The results are shown in Fig. 5. Figure 5a shows the transformed concentration distribution *c(r*) in fringe displacement units as a function of radial position r, obtained from the sedimentation equilibrium interference fringes (inset) and Fig. 5b the corresponding plot of ln*c(r*) vs *r*^2^: the ~ linear nature of this plot is our first indicator of a highly monodisperse solution. The M* extrapolation (Fig. 5c) and hinge point estimation methods (Fig. 5d) from this algorithm point to an overall weight average molar mass for the distribution, *M*_w_ = (47.7 ± 0.1) and (46.4 ± 0.1) kDa, respectively, in very close agreement with the expected value from mass spectroscopy of 45774 Da (Fig. 2). The slightly higher value is possibly a reflection of the presence of a small amount of a higher molecular weight component, as indicated by sedimentation velocity. The slight positive slope of the plot of ‘point’ average weight average molar mass *M*_w_(r), as a function of radial position r, or the corresponding local concentration c(r) would also suggest that (Fig. 5d). So to explore this further, additional analysis was conducted using the MULTISIG algorithm of Gillis and coworkers^33^. This involves a multi exponential fit to the equilibrium distribution which is useful for assaying for the presence of discrete components of different molar mass. The results from this analysis (Fig. 6) also confirm that the VanS is almost completely monomeric with ~99% of material = 45.7 kDa and with <1% of high molar mass material, *M* ~ 200 kDa, completely consistent with the SEDFIT-MSTAR result and the results from sedimentation velocity and mass spectroscopy. The close match between predicted and AUC-obtained molar masses of VanS suggests that detergent was largely removed during the extensive dialysis steps to a level that was hydrodynamically non-significant following protein purification.

### Addition of vancomycin

**Figure 7:**
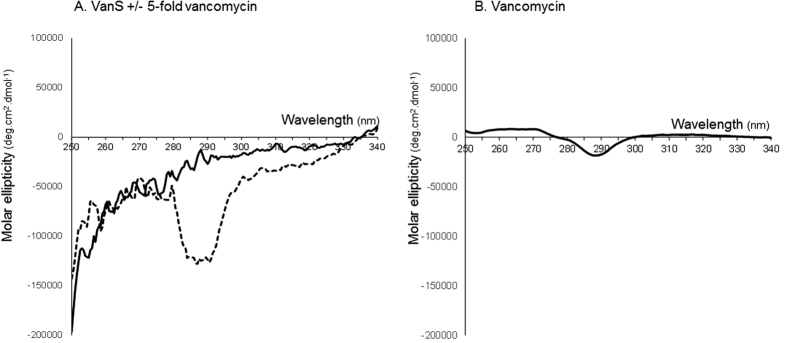
Circular dichroism analysis: molar ellipticity difference spectra of VanS, VanS in the presence of 5-fold vancomycin and vancomycin in the near-UV region at 20.0 °C. (**A**) Solid black line: VanS (9 μM) following subtraction of any contributions from buffer and solvents. Dashed black line: VanS (9.0 μM = 0.41 mg mL^−1^) and vancomycin (45 μM = 0.067 mg mL^−1^) (in phosphate-buffered saline) after subtraction of spectral contributions from vancomycin, buffer and solvents. (**B**) Solid black line: vancomycin (45 μM) following subtraction of any contributions from buffer and solvents. Reaction mixes contained 10 mM HEPES, 20% (v/v) glycerol, 100 mM NaCl and 0.05% n-dodecyl-β-D-maltoside, pH 7.9. Unsmoothed data shown.

Figure 4 also shows the SEDFIT *c(s*) vs *s* analysis of 0.25 mg.ml^−1^ (5.4 μM) VanS in the presence of 19.1 μg/ml (12.8 μM) vancomycin. There is a significant change in the distribution in comparison with VanS by itself. The vancomycin (*M* = 1485 Da) control is currently below the lower limit of resolution of the sedimentation velocity method with current instrumentation (<0.3S) but material (presumably aggregated material) is seen at 0.6 and 0.9S. However its effect on the VanS is to increase the sedimentation coefficient by over 30% from (0.9 ± 0.1)S to (1.2 ± 0.2)S (Table 2). In addition, further higher sedimentation coefficient products appear at ~2.4S, 3.5S and 5.0S that are not present in the highly monomeric VanS by itself. This increase indicates an interaction of the vancomycin with the VanS. VanS-vancomycin interactions were also demonstrated previously in our laboratory and at the Diamond Light Source Ltd, by an alternative biophysical approach - circular dichroism (CD) measurements - and the data is shown in Fig 7. CD spectra obtained in the near-UV region (which interrogate changes in the tertiary structural conformation) show a clear change in the VanS difference spectrum in the presence of five-fold vancomycin, specifically in the 280–300 nm region contributed by Trp and Tyr residues, demonstrating an interaction between VanS and vancomycin (Fig. 7A). The difference spectrum for vancomycin alone is shown for comparison in Fig. 7B^38^.

With the sedimentation velocity plot (Fig. 4), the change in mass caused by the binding of the vancomycin is too small to lead to such a large (33%) change in the sedimentation coefficient by itself. The change is however commensurate with either a ligand induced dimerization and/or a ligand induced compaction of the monomer.

## Discussion

### Monomeric nature of VanS in the absence of ligand

In the absence of vancomycin, the hydrodynamic data are consistent with VanS being in the monomeric state (apart from the ~4% very high molecular mass component). A single peak in a sedimentation velocity experiment is symptomatic of a single solute system. It could also in principle be symptomatic of a rapid monomer-dimer equilibrium^39^ – see for example ref. 40. However, the existence of a dimerization in the absence of ligand can be ruled out on the basis of the sedimentation equilibrium data. The weight average molar mass for the whole distribution *M*_w_ (from Fig. 5c and Fig. 6) is in exact agreement with the monomeric molar mass from mass spectrometry, confirming monomer. Further, if there was a monomer-dimer equilibrium then the point average *M*_w_(*r*) would significantly increase with concentration *c(r)*. There is no evidence for this from Fig 5d.

We conclude therefore that purified intact VanS, in the absence of ligand, is predominantly monomeric under these conditions. VanS preparations and buffers were detergent free, ruling out the possibility that DDM might be responsible for VanS monomer conformation. We also note that the buffer used for the AUC experiments here is similar to that used to purify and maintain His-tagged VanS protein stocks, which were subsequently shown to be active under phosphorylation conditions (Fig. 3).

### Dilute solution conformation of VanS

**Figure 8:**
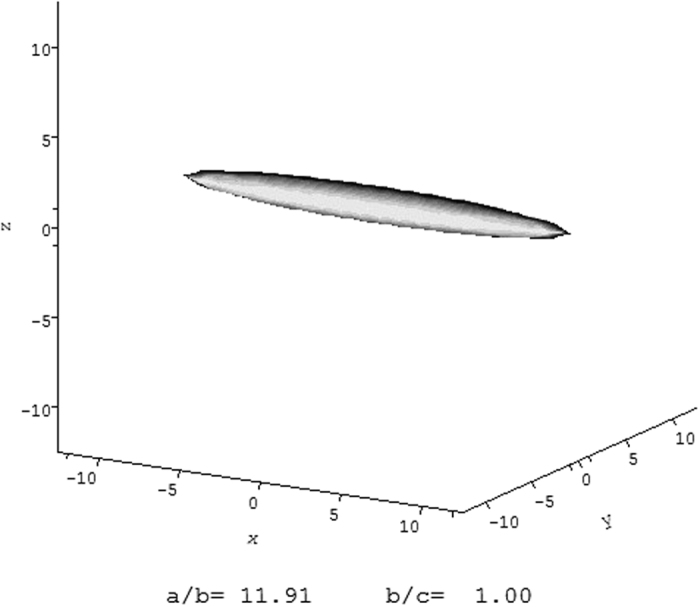
Hydrodynamic shape of the VanS protein from ELLIPS1. The hydrodynamic data is consistent with an asymmetric structure of axial ratio ~(12 ± 2).

It is possible to make an overall assessment of the conformation of VanS monomers, even though the protein is too large for high resolution NMR analysis and the protein has thus far resisted crystallisation. We can estimate the axial ratio of the equivalent hydrodynamic ellipsoid from the sedimentation coefficient and molar mass using the ELLIPS suite of software algorithms of Harding *et al*.^34^, based on reasonable values of the protein hydration δ (of 0.2 to 0.5 g/g). For an *s*_*20,w*_ ~ 2.3S, *M*_*w*_ = 45775 g/mol: these data give a frictional ratio (*f/f*_*0*_) = 1.87 using the routine UNIVERSAL_PARAM. We can then evaluate the Perrin (P) universal shape function of a range of plausible values of the hydration δ from 0.2 to 0.5 g/g (Table 3). This yields an estimate for the axial ratio of the equivalent hydrodynamic (prolate) ellipsoid of revolution of ~(12 ± 2) as shown in Fig. 8. The monomer adopts an extended conformation. Such an extended structure could perhaps reflect the well documented structural architecture and modular nature of the membrane histidine kinase family in general which typically possess multiple domains that usually span the membrane resulting in spatial separation of some signal/ligand perception functions (occurring extracellularly or within/just inside the membrane) from catalytic activity functions (in the cytoplasm)^41^,^42^,^43^. Alternatively, the elongated shape may be a specific feature of VanS that is of functional importance (discussed further below).

### Vancomycin binding and induced changes in VanS

The shift in sedimentation coefficient of the main peak in Fig. 4 on addition of vancomycin, is commensurate with two possible events – (i) a ligand-induced dimerization of the VanS with the additional appearance of 27% higher oligomerization product and/or (ii) a ligand-induced compaction of the monomer, with the appearance of 27% higher oligomerization product. We now consider each of these two possible interpretations of the data in turn.

#### Ligand-induced dimerization

Taking into account the extended conformation of the monomer species, a shift of 33% in *s* can be accounted for by a doubling of the molecular mass i.e. dimerization, allowing for a further small increase due to the vancomycin itself. The resulting *s* value (1.2S, or *s*_20,w_ = 3.1S) would however be too small to be consistent with a side-by side dimerization and too large to be consistent with an end-to end, but would be consistent with an asymmetric binding somewhere between these extremes. Alternatively the *s* of the main peak, 1.2S could be the weighted average of a rapid monomer-dimer equilibrium, with the dimer itself of higher *s* and this would be more commensurate with a side-by-side process. In addition, there is ~27% of high molecular weight material (Table 2) consistent with ligand-induced higher oligomerization. This explanation for the increase in *s* would not be surprising since VanS is predicted to possess a dimerization/phosphoacceptor-type histidine kinase A domain (ncbi.nlm.nih.gov/Structure/cdd) and would be consistent with current mechanisms proposed for downstream signal transmission including the asymmetric piston shift model, scissoring or helical rotation of the two so-called p(eriplasmic) helices into an asymmetric complex or combinations of both of these (reviewed in ref. 41).

#### Ligand-induced compaction of monomer

Alternatively a 33% increase in the *s* value of the main (monomeric peak) could be equally explained by a compaction of the monomer. The increased *s* value for *the same molecular mass* would lead to a new frictional ratio *f/f*_*o*_ = 1.39, and a P value of ~1.22, corresponding to a reduction in axial ratio on ligand binding from 12 to ~5.

To elucidate which of (i) or (ii), or if a combination of the two is responsible, will require further experimentation.

In previous studies of the diverse range of VanS sensor kinase family there has been much debate about the nature of the direct molecular ligands that activate them^23^. It was suggested previously that the VanS proteins of VanA, VanB and other types of vancomycin-resistant strains may all respond to different signals, evidenced in part by the distant relatedness between VanS sensors which share only 16% overall primary sequence identity. In the case of VanA-type strains of clinical enterococci investigated in the present study then, it has been demonstrated here using a direct *in vitro* approach that vancomycin itself interacts directly with VanS. This is of course consistent with *in vivo* studies which showed that vancomycin induces resistance genes in VanA strains^44^,^45^,^46^,^47^,^48^,^49^,^50^,^51^. Direct interaction by the antibiotic itself was also reported for the distantly related VanS_SC_ and VanS_B_ sensors^26^,^27^,^28^. The distant relatedness between these sensors and to the VanS_A_ sensor under study here indicates that the shared feature of these diverse sensors to interact with vancomycin must presumably be exerted through similarities at the tertiary level or alternatively through separate evolution of several different mechanisms to accomplish glycopeptide binding. Clearly future hydrodynamic and spectroscopic studies of these other sensors will help elucidate which of these possibilities might be correct. In any case, the *in vitro* approach used here employing purified protein indicates that binding of the antibiotic to VanS, at least to some extent, does not appear to require additional accessory factors. This study provides a platform for future studies on the elucidation of the strength and stoichiometry of this interaction and the effects of environmental conditions on the interaction, and the relation of the interaction to the oligomeric state, conformation and activity of this important two component system.

## Methods

### Gene cloning

The *vanS* gene of *Enterococcus faecium* BM4147/pFD225 possessing the VanA-type vancomycin resistance operon (NCBI reference sequence WP_002305818.1) was cloned by Djalal Meziane-Cherif (Pasteur Institute) in pTTQ18His as a *NdeI*- and *PstI-*ended PCR-amplified fragment as described previously^30^,^52^ using standard cloning methods^53^ and the following primer pair:

FORWARD: 5′-CGATCATG,AAT,TCG,CAT,ATG,GTT,ATA,AAA,TTG,AAA,AAT,AAA,AAA,AAC-3′

REVERSE: 5′-CGATCCTGCAGC,GGA,CCT,CCT,TTT,ATC,AAC,CAA,GTC,TGG,C-3′

Cloned genes were confirmed by DNA sequencing. The resulting plasmid pTTQ*vanS* possesses *vanS* positioned downstream of the IPTG-inducible *tac* promoter. Recombinant VanS possesses an additional MNSH sequence at the N-terminus and the His-tag sequence _N-_AAGGRGSHHHHHH_-C_ at the C-terminus^35^.

### Overexpression of recombinant His-tagged VanS

*E. coli* BL21 [DE3] harbouring pTTQ*vanS* was cultured aerobically at 37 °C in Luria Bertani (LB) medium containing 100 μg/ml carbenicillin. Induction of VanS expression was achieved by addition of 1 mM isopropyl β-D-1-thiogalactoside (IPTG) to mid-exponential phase cultures followed by incubation for 3 h at 30 °C prior to cell harvesting^30^,^52^.

### Preparation of total *E. coli* BL21 [DE3]/pTTQ*vanS* membranes

Cell pellets were homogenised in 10 mM Tris.HCl, 0.5 mM EDTA, 10% glycerol buffer and lysed using a Constant Systems cell disruptor. Lysates were centrifuged to remove cell debris (18,500 g for 45 min) and membranes prepared as described previously^30^,^35^.

### Purification and final sample preparation for verification of intact VanS protein

Purification of intact his-tagged VanS was undertaken using total mixed membrane preparations of *E. coli* BL21 [DE3]/pTTQ*vanS* induced with IPTG as described previously^30^,^35^,^52^. Detergent-solubilised VanS was bound to bedded nickel-NTA resin columns (Qiagen) as described previously^30^,^35^. The resin was washed with 10 mM HEPES (pH 7.9), 20 mM imidazole, 100 mM NaCl, 0.05% (w/v) *n*-dodecyl-β-D-maltoside, 2 mM β-mercaptoethanol, 20% (w/v) glycerol. VanS was eluted using the same buffer containing 200 mM imidazole. For SDS-PAGE, micellar protein was concentrated with Millipore Centricon filters of 100 MWCO. For AUC experiments, buffer exchange involved two rounds of extensive dialysis with using Spectra/Por^®^ Float-A-Lyzer^®^ G2 (3.5–5 kDa)) columns, using 10 mM HEPES (pH 7.9), 20% (w/v) glycerol, 100 mM NaCl with no added *n*-dodecyl-β-D-maltoside (HGN buffer). In preparation for *in vitro* autophosphorylation assays (which lacked added detergent), purified protein was prepared as described above except that (1) a final concentration of 0.025% DDM was used in all wash steps post-solubilisation; (2) there was no added NaCl in buffers; and (3) the elution buffer comprised 10 mM HEPES pH 7.9, 20% (v/v) glycerol and 0.025% DDM.

### Electrospray ionization mass spectrometry (ESI-MS)

Concentrated protein samples were diluted in 40 mM ammonium acetate to produce samples of at least 5 μM which were analysed by ESI-MS at the Astbury Centre for Structural Molecular Biology, University of Leeds, UK. Calibration of the m/z scale was achieved with a separate injection of horse heart myoglobin.

### N-terminal sequencing

Purified proteins were separated on SDS-polyacrylamide gels, electroblotted onto PVDF membrane and stained with Coomassie Blue G-250. Excised proteins bands were sequenced by Edman degradation at Alta Bioscience Ltd, University of Birmingham, UK.

### Western blotting

Proteins were separated by SDS-PAGE and electroblotted onto PVDF membranes for detection of the hexa-Histidine tag of expressed proteins using the INDIA^TM^ HisProbe-HRP (Perbio Science UK Ltd.) and Supersignal West Pico Chemiluminescent substrate (Pierce) detection system^29^. Chemiluminescence was detected using autoradiography or a gel documentation system.

### Protein determination

Protein determinations were carried out using the BCA assay described in^30^ or by spectrophotometry at 280 nm. Extinction coefficients were calculated using contributions from Trp 5500 and Tyr 1500 residues.

### Autophosphorylation activity assays

Autophosphorylation assays were carried out at 22 °C and 37 °C in buffer with no added detergent (DDM) as described previously^29^,^30^ using 60 pmoles of purified VanS which were incubated for 20 minutes prior to initiation of autophosphorylation reactions. Reactions were initiated by addition of ATP (containing 185 kBq [γ-^33^P] ATP per 20 μl reaction) to a final concentration of 50 μM. Reactions were incubated for 36 min at 37.0 °C or 30 min at 22.0 °C before being stopped as described previously^29^,^30^. Reactions were loaded onto SDS-polyacrylamide gels to separate protein bands. Gels were dried by vacuum at 80 °C for 1 h and analysed by autoradiography.

### Sources of vancomycin

Vancomycin was purchased from Sigma-Aldrich, UK.

### Sedimentation velocity in the analytical ultracentrifuge

Sedimentation velocity experiments were performed in HGN buffer (10 mM HEPES (pH 7.9), 20% (w/v) glycerol, 100 mM NaCl buffer of density = 1.0655 g/ml, viscosity = 0.0209 Poise at 20.0 °C) using a Beckman (Palo Alto, CA, USA) Optima XL-I analytical ultracentrifuge equipped with Rayleigh interference optics and an automatic on-line data capture system. Conventional 12 mm double-sector epoxy cells with sapphire windows were loaded with 400 μL (sample and reference solvent channels) and run at a rotor speed of 40000 rpm at a temperature of 20.0 °C. Concentration profiles and the movement of the sedimenting boundary in the analytical ultracentrifuge cell were recorded using the Rayleigh interference optical system and converted to concentration (in units of fringe displacement relative to the meniscus) versus radial position, *r*^34^. Data were analysed using the c(*s*) vs *s* model incorporated into the SEDFIT analytical algorithm of Dam & Schuck^31^. SEDFIT generates an apparent distribution of sedimentation coefficients in the form of *c(s*) versus *s*, where *s* is the sedimentation coefficient (in Svedberg units S = 10^−13^ sec). This data analysis was followed by the correction to standard solvent conditions - namely the density and viscosity of water at 20.0 °C - to yield s_20,w_ using the algorithm SEDNTERP^54^, which also incorporates the partial specific volume (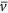) for VanS (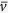 ~ 0.747 mL.g^−1^).

### Sedimentation equilibrium in the analytical ultracentrifuge

Sedimentation equilibrium experiments were also performed using the Beckman (Palo Alto, CA, USA) Optima XL-I analytical ultracentrifuge again using the Rayleigh interference optics and an automatic on-line data capture system, to record equilibrium concentration distribution profiles. The modified long (20 mm) optical path length double-sector titanium cells with sapphire windows were selected and loaded with 130 μL of solution (that had been dialysed against the HGN solvent to constant chemical potential^55^) and a matching amount of reference solvent dialysate in the appropriate channels. An equilibrium speed of 25000 rpm was employed. Because of the much longer duration of a sedimentation equilibrium experiment compared to sedimentation velocity samples were maintained at a low temperature of 7.0 °C and scans were taken once every hour until equilibrium had been reached after approximately 96 hours. Optical records (Rayleigh interference profiles) of the relative concentration distribution of the solute at equilibrium were analysed to give the weight (mass) average apparent molar mass *M*_*w,app*_ using the SEDFIT-MSTAR algorithm^32^. This uses the M* function of Creeth and Harding^37^, together with the hinge point method (evaluation of the point or weight average molar mass at the radial position in the distribution where the local (total macromolecule) concentration *c(r)* = the initial loading concentration, *c*^32^. The use of long path length cells meant that low loading concentrations could be used to give a sufficient signal (~0.326 mg.mL^−1^) for records to be interpreted. At such low concentration, non-ideality effects (which tend to lead to underestimates of the molar mass) may be relatively small and we make the approximation that the apparent weight average molar mass *M*_*w,app*_ is equal to the true weight average molar mass *M*_*w*_^32^. We also use MULTISIG^33^ to estimate the molar mass distribution^56^. This latter routine approximates the concentration distribution at sedimentation equilibrium by a 17-exponential fit: inversion of the exponent coefficients gives then an estimate of the distribution, the weight average molar mass and the polydispersity index (*M*_w_/*M*_n_), where *M*_n_ is the number average molar mass. The method is described in full detail in ref. 33.

### Circular dichroism

Experiments were performed using a nitrogen-flushed instrument on the B23 Synchotron Radiation CD Beamline at the Diamond Light Source, Oxfordshire, UK as described previously^52^.

## Additional Information

**Accession code**: Type-A VanS: UniProtKB – Q06240.

**How to cite this article:** Phillips-Jones, M. K. *et al*. Hydrodynamics of the VanA-type VanS histidine kinase: an extended solution conformation and first evidence for interactions with vancomycin. *Sci. Rep.* **7**, 46180; doi: 10.1038/srep46180 (2017).

**Publisher's note:** Springer Nature remains neutral with regard to jurisdictional claims in published maps and institutional affiliations.

## Figures and Tables

**Table 1:** Sedimentation velocity on 0.25 mg/ml VanS in HGN buffer (10 mM, HEPES, 100 mM NaCl + 20% glycerol).

	*s*_T,b_ (S)	*s*_20,w_ (S)	% w/w
Main component (monomer)	0.9 ± 0.1	2.3 ± 0.1	96%
2^nd^ peak	4.3 ± 0.2	10.9 ± 0.5	4%

Experiments conducted at 20.0 °C at a rotor speed of 40,000 rpm (check). 1S = 10^−13^ sec. *s*_T,b_: the value measured in the temperature and buffer (solvent), not normalized to standard conditions. *s*_20,w_: after correction/normalization to standard solvent conditions – the density and viscosity of water at 20.0 °C.

**Table 2:** Sedimentation velocity of 0.25 mg.mL^−1^ (5.4 μM) VanS in the presence of 19.1 μg/ml (12.8 μM) vancomycin and 20% glycerol (in HGN buffer).

	s_T,b_ (S)	s_20,w_ (S)	% w/w
Main component	1.2 ± 0.1	3.1 ± 0.2	73%
2^nd^ peak	2.4 ± 0.2	6.2 ± 0.5	14%
3^rd^ peak	3.5 ± 0.2	9.1 ± 0.6	6%
4^th^ peak	5.0 ± 0.2	13.0 ± 0.8	7%

Experiments conducted at 20.0 °C at a rotor speed of 40,000 rpm. 1S = 10^−13^ sec.

**Table 3:** The frictional ratio *f/f*_*o*_ for VanS in HGN buffer, corresponding Perrin shape function P for a range of plausible hydration values, and the corresponding axial ratios, *a/b* of the equivalent prolate ellipsoid, from ELLIPS1^34^.

*f/f*_*o*_	Hydration δ	P	*a/b*
1.87	0.2	1.73	13.8
1.87	0.35	1.64	11.9
1.87	0.5	1.56	10.3
